# Drug-loaded mucoadhesive microneedle patch for the treatment of oral submucous fibrosis

**DOI:** 10.3389/fbioe.2023.1251583

**Published:** 2023-09-14

**Authors:** Xian Cheng, Yanqing Yang, Zhengwei Liao, Qiao Yi, Yueying Zhou, Xiaohan Dai, Yanping Liu, Ousheng Liu

**Affiliations:** ^1^ Hunan Key Laboratory of Oral Health Research, Hunan 3D Printing Engineering Research Center of Oral Care, Academician Workstation for Oral-maxilofacial and Regenerative Medicine, Hunan Clinical Research Center of Oral Major Diseases and Oral Health, Xiangya Stomatological Hospital, Xiangya School of Stomatology, Central South University, Changsha, Hunan, China; ^2^ State Key Laboratory of Oral Diseases, West China Hospital of Stomatology, Sichuan University, Chengdu, Sichuan, China; ^3^ Shanghai Key Laboratory of Stomatology, Shanghai Ninth People’s Hospital, College of Stomatology, Shanghai Jiao Tong University School of Medicine, Shanghai, China

**Keywords:** polymeric biomaterials, wet adhesive, microneedle, local drug delivery, oral submucous fibrosis

## Abstract

Oral submucous fibrosis is a chronic, inflammatory and potentially malignant oral disease. Local delivery of triamcinolone to lesion site is a commonly used therapy. The existing methods for local drug delivery include topical administration and submucosal injection. However, in the wet and dynamic oral microenvironment, these methods have drawbacks such as limited drug delivery efficiency and injection pain. Therefore, it is urgently needed to develop an alternative local drug delivery system with high efficiency and painlessness. Inspired by the structure of band-aid, this study proposed a novel double-layered mucoadhesive microneedle patch for transmucosal drug delivery. The patch consisted of a mucoadhesive silk fibroin/tannic acid top-layer and a silk fibroin microneedle under-layer. When applying the annealing condition for the medium content of β-sheets of silk fibroin, the microneedles in under-layer displayed both superior morphology and mechanical property. The mechanical strength of per needle (0.071N) was sufficient to penetrate the oral mucosa. Sequentially, the gelation efficiency of silk fibroin and tannic acid in top-layer was maximized as the weight ratio of tannic acid to silk fibroin reached 5:1. Moreover, *in vitro* results demonstrated the double-layered patch possessed undetectable cytotoxicity. The sustained release of triamcinolone was observed from the double-layered patch for at least 7 days. Furthermore, compared with other commercial buccal patches, the double-layered patch exhibited an enhanced wet adhesion strength of 37.74 kPa. In addition, *ex vivo* mucosal tissue penetration experiment confirmed that the double-layered patch could reach the lamina propria, ensuring effective drug delivery to the lesion site of oral submucous fibrosis. These results illustrate the promising potential of the drug-loaded mucoadhesive microneedle patch for the treatment of oral submucous fibrosis.

## 1 Introduction

Oral submucous fibrosis is a chronic, inflammatory and potentially malignant mucosal disease closely associated with habitual chewing of area nuts (Ray et al., 2019). In recent years, with the expansion of area nut industry, the incidence rate of oral submucous fibrosis has been steadily climbing, arousing it a public health issue in China and the countries in South Asia (Tilakaratne et al., 2016a). The typical clinical symptoms of oral submucous fibrosis include progressive bleaching, burning sensation, increased mucosal stiffness, and the presence of characteristic fibrous bands, which bring about restricted oral movements as well as strenuous chewing and swallowing (Ray et al., 2019). Despite the adverse impact on patients’ life quality, what makes oral submucous fibrosis even more concerning is its tendency for malignant transformation (Wang et al., 2014). Approximately 7.6–13% cases undergo malignant transformation into oral cancer (Guo et al., 2023).

There are multiple administration methods for oral submucous fibrosis, including habit cessation, physical therapy, surgical procedures, and medical treatments (Guo et al., 2023). Among them, medical treatments by local administration of corticosteroids, such as triamcinolone, are most widely used in clinical practice and highly recommended in the latest clinical management guidelines of oral submucous fibrosis (Kerr et al., 2011). Local administration is achieved mainly through two routes: topical administration and submucosal injection (Chole et al., 2012). However, both routes have major hurdles hampering their efficacy. On one hand, topical administration has limited drug residence time due to the mechanical flushing and enzymatic degradation by saliva (Alqahtani et al., 2021). Additionally, the osmotic barrier formed by oral epithelial cells obstructs the penetration and absorption of drugs (Alqahtani et al., 2021). On the other hand, submucosal injection causes significant discomfort, especially for oral submucous fibrosis patients who are more pain-sensitive, greatly reducing the compliance of patients (Cox and Zoellner, 2009). Therefore, alternative approaches should be exploited promptly for superior buccal drug delivery to address the overwhelming clinical demand.

Microneedle system penetrate the epidermal layer in a minimally invasive manner and create microchannels to transport drugs (Lin et al., 2021). With the advantages of high efficiency, minimal invasiveness as well as painlessness, the microneedle system is considered as a promising drug delivery platform in multifarious transdermal/transmucosal applications (Ma and Wu, 2017). Nevertheless, the highly moist and dynamic oral microenvironment limited the application of microneedle system for oral medicine (Macedo et al., 2020). Microneedles could not achieve sufficient adhesion with wet mucosal surfaces only by mechanical anchoring (Guillot et al., 2020). Herein, a novel local delivery microneedle patch with enhanced wet adhesion capacity was proposed for the treatment of oral submucous fibrosis in this study.

As a proof-of-concept, silk fibroin (SF) was selected as the substrate material for microneedles since it has gained a mounting recognition in the field of drug delivery (Zhang et al., 2015; Zhang et al., 2016; Cheng et al., 2021). The predominant advantages of SF are the remarkable cytocompatibility, robust mechanical properties, hypoallergenic features, and tunable biodegradation (Han et al., 2017; Cheng and Yang, 2019; Zhang et al., 2019; Cheng R. H. et al., 2020). But pure SF microneedle system lacks wet adhesion capacity. Marine organisms derived compounds (e.g., pyrogallol and catechol) form wet adhesives by binding with proteins (Fan and Gong, 2021). Pyrogallol-rich tannic acid (TA) displays remarkable wet-resistant adhesion properties, anti-inflammation and wide availability (Gao et al., 2020). Previous studies also reported that TA facilitates the gelation of SF through hydrogen bonding, hydrophobic interactions, and π−π stacking (Jing et al., 2019). The incorporation of TA with SF highly improves the wet-resistant adhesion of SF-based materials (Gao et al., 2020).

**FIGURE1:**
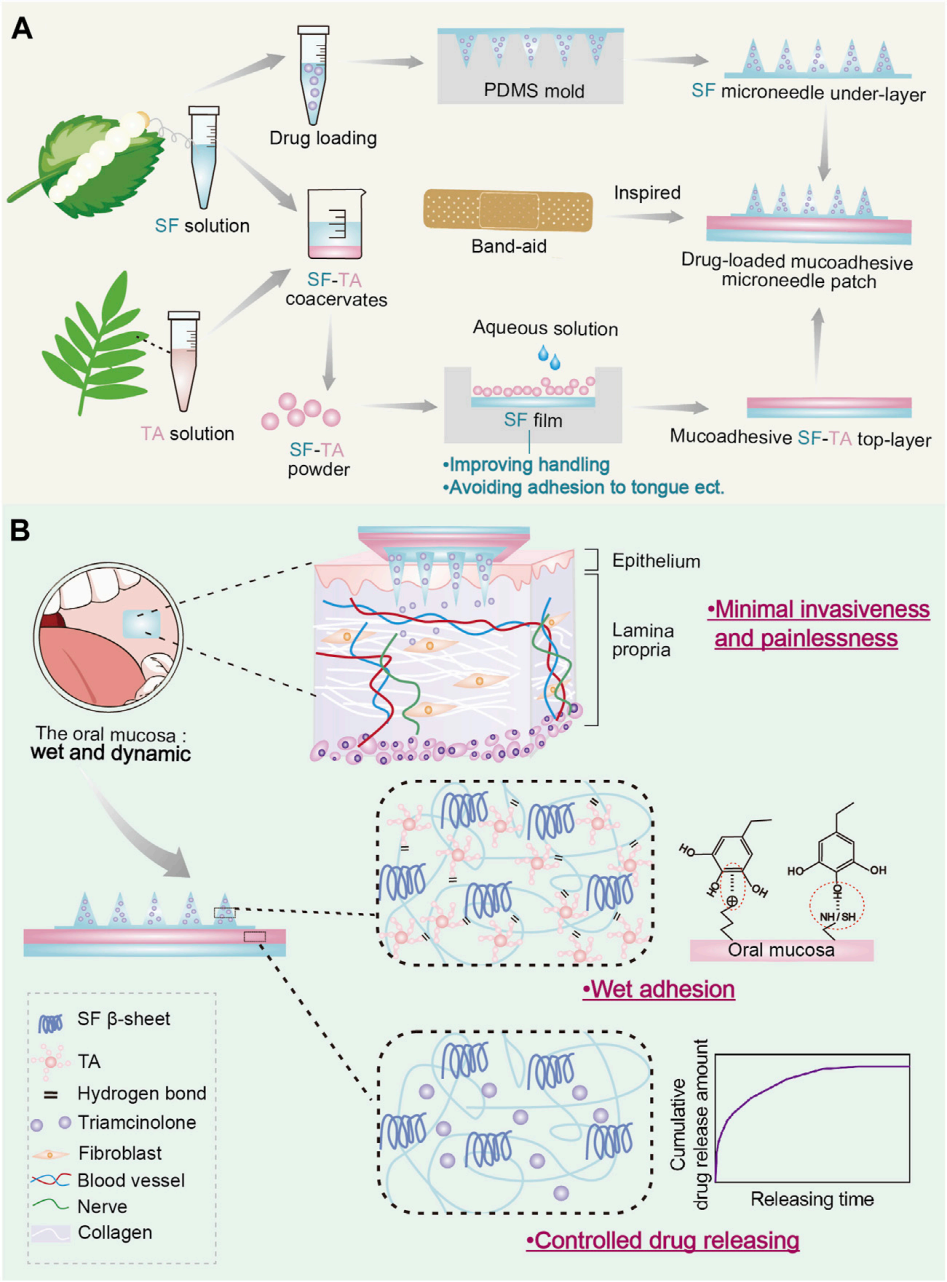
Drug-loaded mucoadhesive microneedle patch for the treatment of oral submucous fibrosis. **(A)** The design and preparation of drug-loaded mucoadhesive microneedle patch. **(B)** Illustration of three major advantages of drug-loaded mucoadhesive microneedle patch in the treatment of oral submucous fibrosis.

Therefore, in this study, inspired by the structure of band-aid, we designed a double-layered mucoadhesive microneedle drug delivery patch comprising a SF microneedle under-layer and a mucoadhesive SF-TA top-layer (Figure 1A). The mucoadhesive top-layer include an adhesive SF-TA gel film to achieve wet adhesion and a non-adhesive silk fibroin handling film to prevent adhesion to surrounding tissues, such as tongue. Firstly, morphology statistics and compression tests were carried out to research the mechanical property of SF microneedle under-layer with different secondary structure conformations. Subsequently, lap-shear tests were conducted to explore the wet adhesion capacity of SF-TA top-layer with different TA/SF weight ratios and the underlying gelation mechanisms between SF and TA were investigated. The interfacial adhesion force between two layers was tested to investigate whether the double-layered microneedle patch would delaminate during usage. Furthermore, the cytocompatibility of the double-layered microneedle patch was explored through co-culturing with fibroblasts. Triamcinolone was selected to characterize the drug releasing capacity of the double-layered microneedle patch. The wet adhesion performance of the double-layered microneedle patch was compared with commercial buccal patches. These results would demonstrate the feasibility of the drug-loaded mucoadhesive microneedle patch as a transmucosal drug delivery system, providing a promising strategy for oral submucous fibrosis (Figure 1B).

## 2 Materials and methods

### 2.1 Preparation of the SF aqueous solution

*Bombyx mori* silk cocoons were provided by the State Key Laboratory of Silkworm Genome Biology of Southwest University, China. The SF aqueous solution was prepared as previously described (Liu et al., 2021a). To be specific, the degumming process was performed in boiled 0.02 M sodium carbonate solution (S111733, Aladdin, China) for 30 min. After being washed with ultrapure water and dried, the extracted silk was dissolved in 9.3 M lithium bromide solution (L108934, Aladdin, China) at 60°C for 4 h and successively dialyzed in ultrapure water for 48 h. Then, the SF solution was centrifugated to remove insoluble residue and diluted to 7 wt%.

### 2.2 Preparation of the SF microneedle under-layer

A polydimethylsiloxane (PDMS) microneedle mold was customed by Taizhou Microchip Technology Co. Ltd. (Taizhou, China). The dimensional parameters are as listed: 10 × 10 mm^2^ with a 10 × 10 conical microneedle array with 300 µm in base diameter, 670 µm in depth and less than 10 µm in tip diameter. The 7 wt% SF solution was first poured into the microneedle mold, followed by being degassed in a vacuum dryer (Muton, Japan) (>0.09 MPa) and centrifugated to remove the bubbles. Subsequently, the drying process (25 °C, 60% humidity) was carried out for 48 h. Finally, to achieve different β-sheet conformation contents in the SF microneedles, the microneedles were annealed in a water vapor-filled vacuum chamber at 4°C or 25°C overnight for low or medium-β group respectively. The immersion in 90% methanol aqueous solution for 10 min was applied as annealing method for high-β group.

### 2.3 Preparation of the SF-TA mucoadhesive top-layer

The SF solution was further diluted to 2 wt%. TA was dissolved in ultrapure water to a concentration of 10 wt%. The SF-TA coacervates were obtained by adding TA solution dropwise to the corresponding SF solution on a magnetic stirrer (MS10-H500-Pro, DWB, China) at 300 rpm with different TA/SF weight ratios. The SF-TA mixtures were stirred and stabilized for 30 min at 300 rpm to allow hydrogen bonding formation between the SF and TA moieties. Subsequently, the SF-TA coacervates were settled down for 30 min and washed with ultrapure water to remove the residual unbound TA. Afterwards, the SF-TA coacervates were collected and lyophilized in a freeze-drying machine (ZLGJ-8, Labconco, China) for 48 h. Finally, the lyophilized SF-TA coacervates were ground in a ball grinder (PM100, Retsch, Germany) to obtain the SF-TA lyophilized powder and stored at −20°C.

Another cylindrical concave PDMS mold (Taizhou Microchip Technology Co. Ltd., Taizhou, China) was used with an internal groove (20 × 20 × 0.2 mm^3^). 7 wt% SF solution was poured into the groove, followed by being degassed in a vacuum dryer (Muton, Japan) (>0.09 MPa) and centrifugated to remove the bubbles. Subsequently, the drying process (25°C, 60% humidity) was carried out for 48 h. The mold was then put into the water vapor-filled vacuum chamber at 25°C overnight for annealing to prepare the non-adhesive SF handling film. The SF-TA lyophilized powder was added onto the annealed SF handling film in the mold. By adding a small amount of ultrapure water, the SF-TA lyophilized powder restored into a mucoadhesive SF-TA gel film (Gao et al., 2020). The non-adhesive SF handling film and the mucoadhesive SF-TA gel film quickly bonded into an integrity, named SF-TA mucoadhesive top-layer.

### 2.4 Preparation of the double-layered mucoadhesive microneedle patch

The SF microneedle under-layer was put onto the center of the SF-TA mucoadhesive top-layer with the microneedle tips upward (Figure 1A). The double-layered mucoadhesive microneedle patch was successfully fabricated by the quick and solid bonding between the SF and SF-TA interface.

### 2.5 Fourier transform infrared spectroscopy

Fourier transform infrared spectroscopy analysis was performed using attenuated total reflectance infrared spectroscopy (ALPHA Ⅱ, Bruker, Germany). For calculation of the β-sheet conformations in the SF microneedle under-layer, the amide I region (1595 cm^–1^ ∼ 1705 cm^−1^) was determined by Fourier self-deconvolution using OMINIC software and subsequent curve fitting by Origin software according to a previously reported method (Lu et al., 2011) (n = 3).

### 2.6 Differential scanning calorimetry

Differential scanning calorimetry measurement was performed using a differential scanning calorimeter (DSC3, Mettler, Switzerland) under nitrogen atmosphere. The SF-TA coacervates (TA/SF weight ratio = 5:1) (∼10 mg) were encapsulated in a perforated aluminum pan and the differential scanning calorimetry curve was obtained at a heating rate of 10°C/min within the temperature range 25∼250°C.

### 2.7 Morphology of the microneedles

The microstructure of microneedles was imaged by a Field Emission Scanning Electron Microscopy (MIRA3 LMH, TESACN, China) at 5 kV after being sputter-coated with platinum.

An optical microscopy (S APO, Leica, Germany) was used for the observation and statistics of microneedles’ morphology. Specifically, the microneedle patches were fixed on glass slides with the tips upward, positioned at a 45° incline on the microscope stage. All 100 microneedles of each patch were observed under the optical microscopy. The counts of bent tips and microneedles deviating over 20° were recorded (n = 3). Tip bending ratio and microneedle tilt ratio were calculated by using the following equations respectively:

$$\text{Tip bending ratio }\left(\%\right)=\frac{n1}{n}\times 100\%$$

$$\text{Microneedle tilt ratio }\left(\%\right)=\frac{n2}{n}\times 100\%$$

where n1 and n2 represent the counts of bent tips and microneedles deviating over 20°, respectively. *n* refers to the total number of microneedles being analyzed.

The microneedle patches were immersed in the 1.5 mM DAPI solution for 5 min under low-light conditions. The immersed patches were placed upside down on glass slides positioned parallelly on the microscopy stage. A fluorescence microscope (DM500, Leica, Germany) was used to observe the morphology of microneedles.

### 2.8 Mechanical property of the microneedles

Compression tests were carried out to evaluate the mechanical strength of microneedles by a universal testing machine (MTS Insight, MTS, United States of America). Briefly, the SF microneedle under-layer was attached to a rigid platform positioned horizontally with the tips upward. The test station sensor approached the microneedles in the vertical direction at a speed of 10 mm/min. The initial distance between the sensor and microneedle tips was 1 cm. Displacement and force were recorded when the sensor first touched the microneedle tips.

### 2.9 Adhesion strength of the SF-TA mucoadhesive gel film in the top-layer

To examine the adhesive strength of SF-TA top-layer, a lap-shear test was conducted by the universal testing machine (MTS Insight, MTS, United States of America). The porcine buccal mucosa was purchased from slaughterhouse and cut into the size of 3 × 1 × 0.5 cm^3^. SF-TA mucoadhesive gel film was restored from lyophilized powder followed by the same method as described in the mucoadhesive SF-TA top-layer preparation part above. This mucoadhesive gel film was put between two mucosa tissues and the bonding area was 1 × 1 cm^2^. Tensile speed was at a rate of 10 mm/min and the adhesive strength (kPa) was calculated by dividing the load (N) by bonding area (m^2^) (n = 4).

### 2.10 Rheological test of the SF-TA mucoadhesive gel film in the top-layer

The rheological properties were tested using a rotating rheometer (Rheo Compass, Anton Paar, China) with a 25 mm diameter rotating plate. The storage modulus (G′) and loss modulus (G″) were monitored by frequency sweeps from 30 to 1 Hz at a strain of 0.1% in the linear viscoelastic range at 37°C. To characterize the effect of temperature on the rheological properties, the temperature was gradually raised from 0°C to 60°C at 1 Hz.

### 2.11 Cytocompatibility of double-layered mucoadhesive microneedle patch

Fibroblasts were obtained from American Type Culture Collection (NIH3T3 cells, mouse embryonic fibroblastic cell line). Cells were cultured in DMEM medium (C3103-0500, Vivacell, United States) containing 10% bovine serum albumin (C2910-0500, Vivacell, United States) and 1% penicillin and streptomycin solution (C3420-0100, Vivacell, United States) in a cell incubator at 37°C and 5% CO_2_.

The samples were immersed in DMEM medium for 1 day to obtain extraction solutions according to ISO 10993 guidance. Specifically, the extract ratio was 0.1 g/mL (the weight of samples/the volume of extract media). The extraction solution was sterilized using a filter. Fibroblasts were seeded in 24-well plates with the cell density of 1.0 × 10^4^ cells/cm^2^ and cultured in DMEM medium, DMEM medium with 5% DMSO and extracts (all containing 10% bovine serum albumin) for 24 h, respectively.

Cytocompatibility was evaluated using Live/dead assay kit (40719ES60, Yeason, China) following the manufacturer instructions. The cell morphology was observed by immunofluorescent staining of cytoskeleton using rhodamine phalloidine (40734ES80, Yeason, China). Cells were visualized using a fluorescent microscope (DM500, Leica, Germany).

The CCK8 assays (40203ES60, Yeason, China) were used to assess cell proliferation quantitatively after incubating for 1, 2, and 3 days (n = 4). The CCK8 results were tested spectrophotometrically (Bio-Tek FL600 microplate fluorescence reader, Biotek, United States of America) according to the manufacturer instructions.

### 2.12 Drug loading and releasing

10 mg/mL triamcinolone solution was added to 7 wt% SF solution by the volume ratio of 1:10. The obtained drug-loaded SF solution was used to prepare microneedles following the microneedle preparation method mentioned above. The drying process (25°C, 60% humidity) was carried out for 48 h. The mold was then put into the water vapor-filled vacuum chamber at 25°C overnight for annealing. Fourier transform infrared spectroscopy analysis was performed to confirm the loading of triamcinolone using attenuated total reflectance infrared spectroscopy (ALPHA Ⅱ, Bruker, Germany).

The drug-loaded SF microneedle was assembled with mucoadhesive SF-TA top-layer to prepare the drug-loaded mucoadhesive microneedle patch. The patches were immersed in 1 mL PBS solution (SH30256.01, Cytiva, United States) and put in a shaker (37°C, 60 rpm). 500 μL supernatant was collected at each time point and another 500 μL fresh PBS solution was added. The accumulative release rate of triamcinolone (%) was calculated with the following equation: accumulative release (%) = (total amount of triamcinolone released)/(total loading amount) × 100%.

### 2.13 *ex vivo* insertion tests

The Sprague-Dawley rats aged 8 weeks (250 ± 10 g) were provided by Hunan SJA Laboratory Animal Co., Ltd. (Hunan, China). The rats were anesthetized using a standard ketamine and xylazine mix (ketamine: 90–120 mg/kg; xylazine: 10 mg/kg) and then sacrificed using cervical dislocation method. The mucoadhesive microneedle patches were applied on the oral mucosa of rats for 5 min. The microneedles treated oral mucosa was fixed in 4% paraformaldehyde fixation solution (C01-06002, Bioss, China) for 24 h, followed by paraffin embedding and H&E staining. The staining results were observed by a brightfield microscope (DM500, Leica, Germany).

### 2.14 Statistical analysi*s*

All data was expressed as means ± standard deviation (SD). By using GraphPad Prism 7.0, significant differences between statistical data in different groups were assessed via *t*-test (two groups) or one-way ANOVA followed by Tukey *post hoc* test used for multiple comparisons (no less than three groups).

## 3 Results and discussion

### 3.1 Fabrication and characterization of the SF microneedle under-layer

**FIGURE 2:**
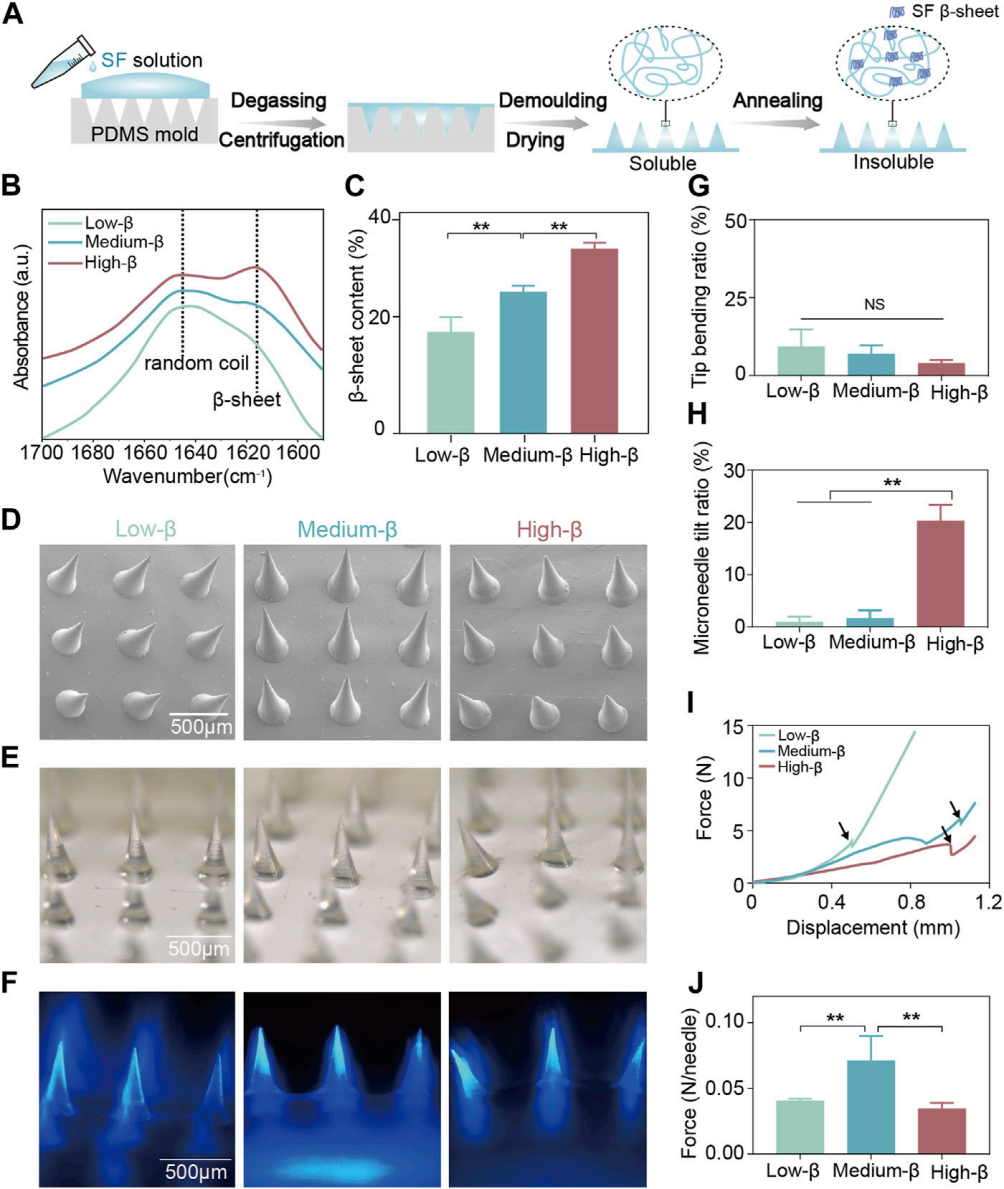
Fabrication and characterization of SF microneedle under-layer. **(A)** Schematic illustration of the preparation process of SF microneedle under-layer. **(B)** The Fourier transform infrared spectroscopy spectra of the amide I region (between 1695 and 1595 cm^−1^) of SF microneedle under-layers and **(C)** the β-sheet contents calculated by Fourier self-deconvolution from these spectra. **(D–F)** The scanning electron microscopy, optical microscopy, and fluorescence microscopy images of SF microneedle under-layers (blue: DAPI). **(G,H)** Quantitative analysis of tip bending ratio and microneedle tilt ratio of SF microneedle under-layers by optical microscopy. **(I)** Force-displacement curves of SF microneedle under-layers. The fracture force point was labeled with an arrow. **(J)** Fracture force per needle of SF microneedle under-layers. Error bars represent standard deviation.

Figure 2A illustrates the preparation process of the SF microneedle under-layer. Since SF microneedles are used in the moist oral environment, an essential annealing step during preparation is to partially transform the SF molecules from a random coil to stable β-sheet conformation to render the SF water-insoluble (Long D. et al., 2021; Long et al., 2021b; Cheng et al., 2021). Three kinds of SF microneedles were constructed with low, medium and high β-sheet contents of 18.07% (low-β group), 24.86% (medium-β group) and 34.65% (high-β group), respectively (Figures 2B, C). Depending on the various conformation conversion methods, the β-sheet contents in SF materials range from ∼15% to ∼60% in literature (Lu et al., 2011; Liu et al., 2021b; Long et al., 2021c). Our prepared SF microneedles contained three representative β-sheet contents within this range.

The scanning electron microscopy, optical microscopy, and fluorescence microscopy (Figure 2D–F) examined the morphologies of three SF microneedle groups of different β-sheet contents. All three groups had uniformly distributed conical microneedles with sharp tips (Figure 2D–F). The tip bending ratios of three groups were comparable and all below 10% (Figure 2G), indicating their excellent tip morphologies. However, the microneedle tilt ratio of high-β group (20.33%) was significantly higher than that of low-β and medium-β groups (Figure 2H). This may happen when a large amount of β-sheet structure formation induced the shrinkage of SF microneedles, changing the vertical alignment between microneedles and bases (Lee H. et al., 2020).

To ensure oral transmucosal delivery, the SF microneedles must be qualified with adequate mechanical properties to penetrate oral mucosa (Lin et al., 2021) The force-displacement curve in Figure 2I showed that the force increased slightly corresponding to the warpage of the microneedles in the initial stage. The fragments of the microneedle body collapsed and were tightly compacted to create a sudden drop and a following upsurge curve. This trend was in coincidence with previous research (Lin et al., 2021). The turning point (labeled with arrows in Figure 2I) was chosen as the fracture force point. As shown in Figure 2J, the fracture force per needle for medium-β group (0.071 N) was higher than that for low-β (0.041 N) and high-β (0.034 N) groups. According to Römgen’s quantitative study, microneedles with a 10 mm tip diameter require the minimum force of 0.05 N per needle to penetrate the stratum corneum (Römgens et al., 2014). Only the mechanical strength of microneedles in medium-β group was sufficient to penetrate the oral mucosa. Previous studies reported that β-sheet content determines the mechanical property of SF materials, which may result from the fact that the abundant hydrogen bonds in β-sheet effectively dissipate the molecular stick-slip deformation during stress loading (Keten et al., 2010; Yin et al., 2018). As the β-sheet content increases, the stiffness of the SF-based materials is improved (Long D. et al., 2021; Cheng et al., 2023). On the other hand, the mechanical properties of microneedles are also correlated with the number of upright microneedles (Lee K. J. et al., 2020). This may explain that medium-β group had the highest mechanical strength because it was a compromise between morphology and stiffness. Therefore, medium β-sheet content was selected as the optimum SF annealing condition in the following experiments.

### 3.2 Fabrication and characterization of the SF-TA mucoadhesive top-layer

**FIGURE 3:**
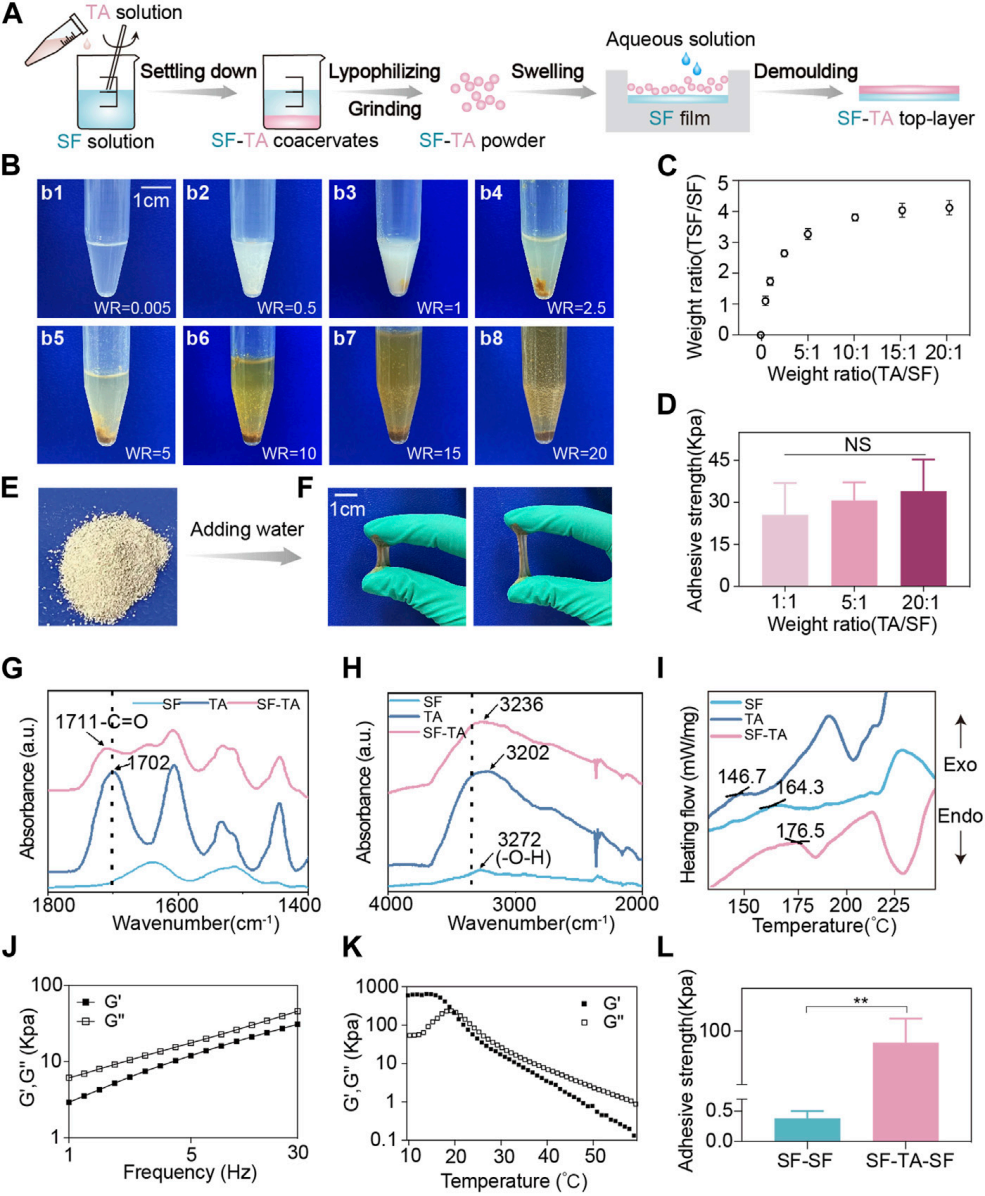
Fabrication and characterization of mucoadhesive SF-TA top-layer. **(A)** Schematic illustration of the preparation process of mucoadhesive SF-TA top-layer. **(B)** Digital photographs of SF-TA coacervates (WR: weight ratio of TA/SF). **(C)** The yield of SF-TA coacervates (SF-TA/SF weight ratio) as a function of the SF/TA weight ratio. **(D)** The adhesive strength of mucoadhesive SF-TA top-layers. **(E)** Digital photograph of SF-TA lyophilized powder. **(F)** SF-TA lyophilized powder restored into SF-TA coacervates with stretchability after adding water. **(G,H)** The Fourier transform infrared spectroscopy spectra of SF, TA and SF-TA coacervates. **(I)** The differential scanning calorimetry curves of SF, TA, and SF-TA coacervates. **(J)** Storage modulus (G′) and loss modulus (G″) of SF-TA coacervates in frequency sweep experiment at 37°C. **(K)** The effect of temperature on G′ and G″ of SF-TA coacervates. **(L)** The interfacial adhesion of SF-TA film with SF film. Error bars represent standard deviation.

The mucoadhesive SF-TA top-layer was synthesized by simply adding TA solution to SF solution. SF-TA coacervates formed the instant TA was added and settled down subsequently (Figure 3A). In previous study, excessive TA was added to SF solution to prepare hydrogels (Gao et al., 2020). From a production perspective, we investigated the quantitative relationship between the TA/SF weight ratio and the yield of SF-TA coacervates. The digital photographs displayed the color of the mixture underwent a gradual darkening from white to brown and the inflection point was TA/SF weight ratio of 5:1 (Figure 3B). The inflection point implied the reaction between TA and SF reached saturation because excessive TA appeared as brown (Gao et al., 2020). This phenomenon also aligned with the quantitative yield data of SF-TA coacervates, which showed the yield ceased to increase when the TA/SF weight ratio ≥5:1 (Figure 3C). This is likely due to saturation of non-covalent hydrogen bonding between SF and TA when TA/SF weight ratio ≥5:1 (Yu et al., 2019). Meanwhile, there was no significant difference in adhesive strength (∼30N) among representative SF/TA weight ratios (Figure 3D). This may result from the fact that the washing process removed excessive TA or SF, leading to a similar conformation in the SF-TA coacervates. The adhesive strength was comparable or even higher than other reported wet adhesives (Hu et al., 2021). Hence, SF-TA coacervates had remarkable wet adhesion property and the extent of reaction between SF and TA had insignificant effect on it. Considering both yield and wet adhesive strength of SF-TA top-layer, 5:1 was considered to be the optimum TA/SF weight ratio and was used in subsequent experiments.

The obtained SF-TA coacervates were processed into freeze-dried powder, which is convenient for storage (Figure 3E). After spraying a small amount of aqueous solution, the powder restored into gel with good stretchability and adhesion property (Figure 3F). The interactions between SF and TA were investigated by Fourier transform infrared spectroscopy and differential scanning calorimetry. The Fourier transform infrared spectroscopy measurement showed that the peak of -C=O vibration in TA shifted from 1702 cm^-1^ (free TA) to 1711 cm^-1^ (TA in SF-TA coacervates) (Figure 3G). A shift of -OH from 3272 cm^-1^ for pure SF to 3235 cm^-1^ was also observed with the addition of TA (Figure 3H). These two wavenumber shifts were consistent with previous studies, indicating that the -OH and -C=O in TA participated in the formation of hydrogen bonding with SF (Jing et al., 2019). In terms of differential scanning calorimetry test, the pure SF displayed the endothermic phase transition at 164.3°C (T_g_) (Figure 3I), reflecting the glass transition of SF chains during heating (Gao et al., 2020). The introduction of TA resulted in an increase of T_g_ to 176.5°C in SF-TA coacervates (Figure 3I), implying the strong hydrogen bonding between SF and TA (Gao et al., 2020). All these results indicate that TA may quickly bind to SF through non-covalent interactions such as hydrogen bonding, enabling them to self-assemble layer by layer into SF-TA coacervates (Bai et al., 2020).

Then the viscoelasticity of SF-TA coacervates was investigated. The loss modulus (G″) was always higher than the storage modulus (G′) at different frequencies (Figure 3J), indicating that SF-TA coacervates had sufficient flowability on the surface of the attached substrate (Zare and Rhee, 2019). As the temperature increased, the G′ and G″ values rapidly decreased and a transition from rubbery state into viscous flow state was observed at 22°C (Figure 3K). That is because the elevated temperature would break the hydrogen bonds and then reduce the cohesion strength of SF-TA coacervates (Gao et al., 2020). In order to improve handling and prevent adhesion to surrounding oral tissues such as tongue, the SF-TA gel film was combined with a non-adhesive SF handling film to form the mucoadhesive SF-TA top-layer (Figure 3A). The SF-TA and SF showed an enhanced interfacial adhesion (94.54 kPa) compared to SF-SF interface (0.38 kPa) (Figure 3L) and other reported double-layered hydrogel interfaces (Li et al., 2018; Hao et al., 2023). This could be attributed to two possible reasons. On one hand, the surface tension and polarity of two SF-based materials were approximate to avoid voids and defects in the interface, resulting in an improved interfacial adhesion (Zheng et al., 2022). On the other hand, free phenolic hydroxyl groups from SF-TA coacervates interact with amino and sulfydryl groups in SF by hydrogen bonding (Zhang et al., 2023). Hence, the double-layered mucoadhesive microneedle patch would remain an integrity and not delaminate during usage.

### 3.3 Cytocompatibility, drug releasing and wet adhesion of the double-layered mucoadhesive microneedle patch

**FIGURE 4:**
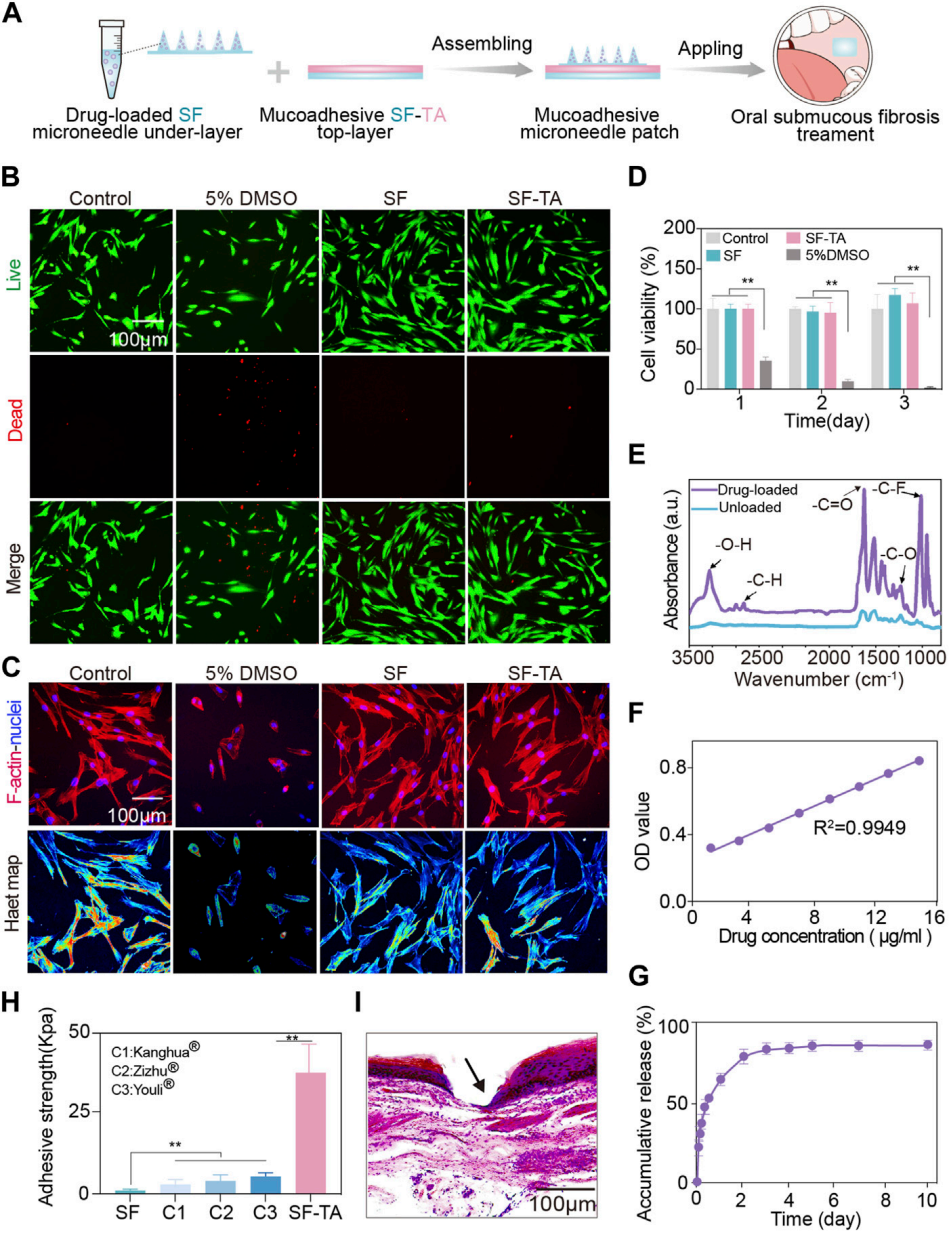
Cytocompatibility, drug releasing and wet adhesion of double-layered mucoadhesive microneedle patch. **(A)** Schematic illustration of the preparation process of double layered mucoadhesive microneedle patch. **(B)** The fluorescence microscopy images of live (green) and dead (red) staining of fibroblasts. **(C)** The immunofluorescent images of F-actin (red) and nucleus (blue) of fibroblasts as well as the corresponding heatmap of F-actin. **(D)** CCK8 results of fibroblasts in 3 days. **(E)** The Fourier transform infrared spectroscopy spectra of triamcinolone-loaded microneedle patch. **(F)** Standard curve of triamcinolone. **(G)** The drug releasing curve of triamcinolone in 10 days. **(H)** The adhesive strength for SF, SF-TA and three commercial buccal films. **(I)** H&E staining images of oral mucosa tissue penetration treated by double-layered mucoadhesive microneedle patch. The arrow indicates puncture sites. Error bars represent standard deviation.

The double-layered mucoadhesive microneedle patch was prepared by integrating the mucoadhesive SF-TA top-layer with the SF microneedle under-layer (Figure 4A). The SF-TA top-layer can firmly adhere to the oral mucosa while SF microneedle under-layer facilitates transmucosal drug delivery. To evaluate the cytocompatibility of the patch, cell survival, viability and morphology were assessed by co-culturing fibroblasts with the extraction solutions derived from two constituents—SF and SF-TA. The fibroblasts in SF and SF-TA groups manifested minimal cell death (Figure 4B). F-actin fluorescence images and the corresponding heat maps illustrated the typical spindle-shaped morphology of fibroblasts in SF and SF-TA group compared to the irregular cell morphology caused by cytotoxic 5% DMSO (Nguyen et al., 2020) (Figure 4C). Furthermore, higher cell viability was observed in both SF group (125.4% ± 19.8%) and SF-TA group (129.0% ± 15.1%) after 3 days of co-culturing (Figure 4D). This may attribute to the regeneration effect of SF (Cheng X. et al., 2020; Long et al., 2021b; Cheng et al., 2023). These results were congruent with earlier studies and confirmed the superior cytocompatibility of the double-layered mucoadhesive microneedle patch (Yu et al., 2019; Pan et al., 2022).

Triamcinolone is a small molecular corticosteroid extensively used in oral submucous fibrosis treatment for anti-inflammation (Tilakaratne et al., 2016b). It was incorporated into SF solution and formed the microneedle under-layer of the patch, referred to as drug-loaded group (Figure 4A). Compared to the pure SF group, the drug-loaded group showed typical infrared absorption bands of triamcinolone at 3285 cm^−1^ and 1624 cm^−1^, which were associated with the stretching vibration of hydrogen bonded hydroxyl and carbonyl group at aliphatic ester bonds, respectively. Other infrared absorption bands of triamcinolone were also observed at 1236 cm^−1^ and 1215 cm^−1^, indicating asymmetric axial deformation of C–O–C bond in aliphatic esters and stretching vibration of C-F, respectively (García-Millán et al., 2017) (Figure 4E). These results verified the loading of triamcinolone in drug-loaded group. The subsequent drug releasing amount was calculated based on the standard curve of triamcinolone (Figure 4F). Triamcinolone showed a controlled release and the accumulative releasing amount reached 88.37% in 7 days (Figure 4G). The unreleased portion of approximately 10% triamcinolone might be caused by losses during demolding. The controlled release of small molecular drug triamcinolone may be attributed to the hydrophobic effects of the aliphatic esters and C-F groups in triamcinolone (Qu et al., 2023). In clinical practice, submucosal injections are administered every 1–2 weeks (Loganathan and Ranganathan, 2023). Hence, the drug releasing time of our patch partially accords with the treatment course. However, how to achieve more sustained and precise release should be further studied. To demonstrate the broad drug delivery capability of the patch, a macromolecular model drug BSA was chosen as a model drug. The drug loading and releasing results were displayed in Supplementary Figure S1.

Furthermore, the wet adhesion capacity of double-layered mucoadhesive microneedle patch was compared with three commercial buccal patches, including Kanghua film^®^ (containing polyvinyl alcohol, glycerin, crystallose, etc.), Zizhu film^®^ (containing glycerin, polyvinyl alcohol, povidone, etc.), and Youli film^®^ (Carbomer, hydroxypropyl methyl cellulose, betadex, etc.). The double-layered mucoadhesive microneedle patch exhibited enhanced wet adhesion (37.74 kPa) on oral mucosal tissues, approximately 7 times higher than those commercial oral patches (<5 kPa) (Figure 4H). When compared with other reported wet adhesives, the double-layered mucoadhesive microneedle patch demonstrated equivalent or even superior wet adhesive strength (Wu et al., 2023). The strong wet adhesion may derive from the non-covalent bonding between the patch and oral mucosal tissue. The free phenolic hydroxyl groups in the patch interact with the amino and sulfhydryl groups in the oral mucosal tissue through hydrogen bonding (Peng et al., 2020). Meanwhile, the cations on oral mucosa would establish cation - π bonding with the pyrogallol or catechol groups from SF-TA coacervates in the patch (Xie et al., 2019). The histological image showed the double-layered mucoadhesive microneedle patch effectively penetrated the epithelial layer of *ex vivo* oral mucosal tissues and reached the lamina propria. Since the histopathologic features of submucosal fibrosis are juxta-epithelial inflammation and intensified collagen in lamina propria and submucosa layer (Ray et al., 2019), the patch enabled drug delivery to the lesion site of oral submucous fibrosis. Based on the above experimental results, the double-layered mucoadhesive microneedle patch has a promising application prospect for transmucosal drug delivery in oral submucous fibrosis treatment.

## 4 Conclusion

In this study, a mucoadhesive microneedle patch for local delivery in oral mucosa was proposed inspired by the structure of band-aid. The added value of the proposed design was demonstrated by developing a novel double-layered microneedle patch, which consisted of a mucoadhesive silk fibroin/tannic acid top-layer and a silk fibroin microneedle under-layer. When applying the annealing condition for the medium content of β-sheets of silk fibroin, the microneedles in under-layer displayed both superior morphology and mechanical property. The mechanical strength of per needle (0.071 N) was sufficient to penetrate the oral mucosa. Sequentially, the gelation efficiency of silk fibroin and tannic acid in top-layer was maximized as the weight ratio of tannic acid to silk fibroin reached 5:1. Moreover, *in vitro* results demonstrated the double-layered patch possessed undetectable cytotoxicity. The sustained release of triamcinolone was observed from the double-layered patch for at least 7 days. Furthermore, compared with other commercial buccal patches, the double-layered patch exhibited an enhanced wet adhesion strength of 37.74 kPa. In addition, *ex vivo* mucosa tissue penetration experiment confirmed that the double-layered patch could reach the lamina propria, ensuring effective drug delivery to the lesion site of oral submucous fibrosis. These results illustrate the promising potential of the drug-loaded mucoadhesive microneedle patch for the treatment of oral submucous fibrosis.

## Data Availability

The raw data supporting the conclusion of this article will be made available by the authors, without undue reservation.
