# Supplementary material for: Drug-loaded mucoadhesive microneedle patch for the treatment of oral submucous fibrosis
Source: Front Bioeng Biotechnol. 2023 Sep 14;11:1251583. doi: 10.3389/fbioe.2023.1251583 (PMC10537940; doi:10.3389/fbioe.2023.1251583)
Supplement: Supplementary file 1 [file DataSheet1.docx]

Supplementary Material

# Supplementary Figures


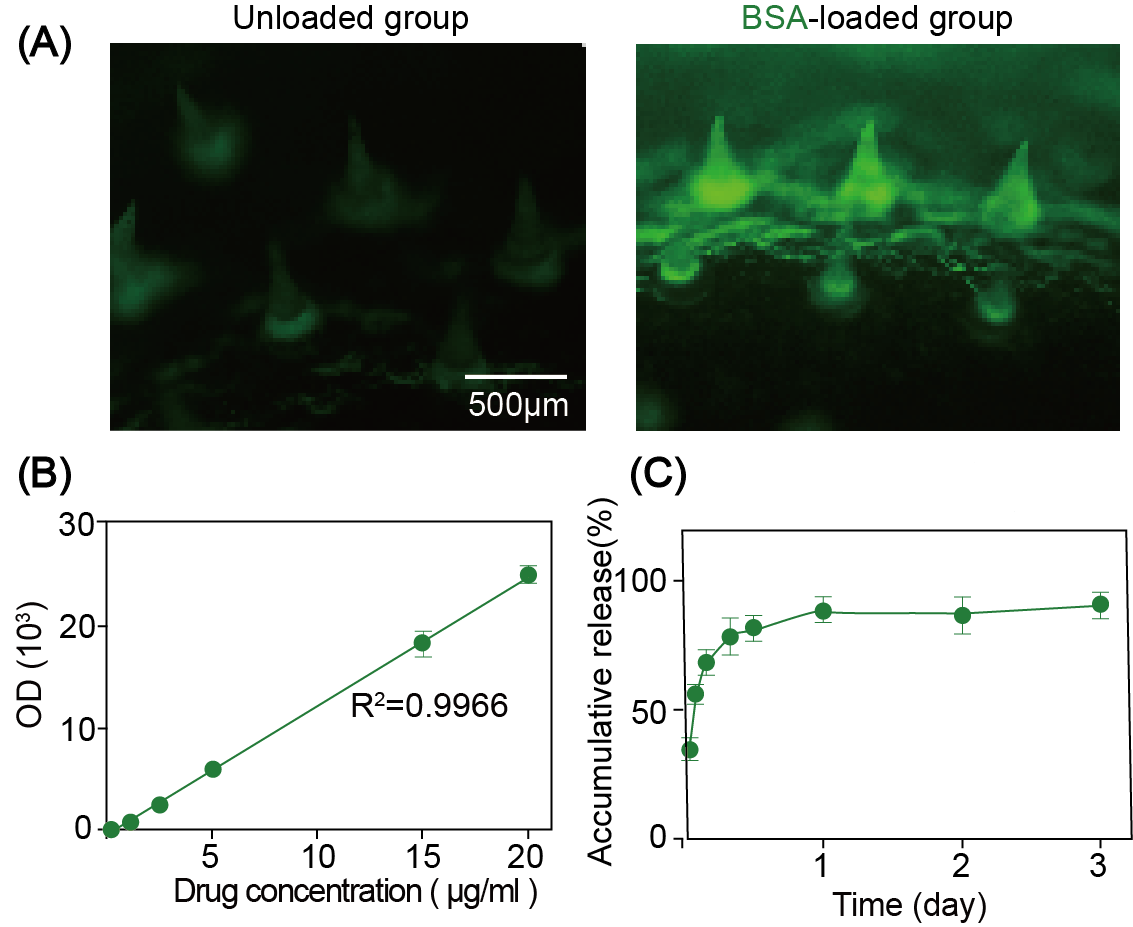


**Supplementary Figure 1.** **BSA loading and releasing of double-layered mucoadhesive microneedle patch.** **(A)** The fluorescence microscopy image of unloaded double-layered mucoadhesive microneedle patch (unloaded group) and BSA-loaded double-layered mucoadhesive microneedle patch (BSA-loaded group). Green fluorescence: FITC. **(B)** Standard curve of BSA. **(C)** The drug realsing curve of triamcinolone in 3 days. Error bars represent one standard deviation.
